# Real-time Trajectory Planning and Tracking Control of Bionic Underwater Robot in Dynamic Environment

**DOI:** 10.34133/cbsystems.0112

**Published:** 2024-05-09

**Authors:** Feng Ding, Rui Wang, Tiandong Zhang, Gang Zheng, Zhengxing Wu, Shuo Wang

**Affiliations:** ^1^ State Key Laboratory of Multimodal Artificial Intelligence Systems, Institute of Automation, Chinese Academy of Sciences, Beijing, China.; ^2^ School of Artificial Intelligence, University of Chinese Academy of Sciences, Beijing, China.; ^3^ Centrale Lille, CRIStAL-Centre de Recherche en Informatique Signal et Automatique de Lille, University of Lille, 59000 Lille, France.; ^4^ Center for Excellence in Brain Science and Intelligence Technology, Chinese Academy of Sciences, Shanghai, China.

## Abstract

In this article, we study the trajectory planning and tracking control of a bionic underwater robot under multiple dynamic obstacles. We first introduce the design of the bionic leopard cabinet underwater robot developed in our lab. Then, we model the trajectory planning problem of the bionic underwater robot by combining its dynamics and physical constraints. Furthermore, we conduct global trajectory planning for bionic underwater robots based on the temporal-spatial Bezier curves. In addition, based on the improved proximal policy optimization, local dynamic obstacle avoidance trajectory replanning is carried out. In addition, we design the fuzzy proportional-integral-derivative controller for tracking control of the planned trajectory. Finally, the effectiveness of the real-time trajectory planning and tracking control method is verified by comparative simulation in dynamic environment and semiphysical simulation of UWSim. Among them, the real-time trajectory planning method has advantages in trajectory length, trajectory smoothness, and planning time. The error of trajectory tracking control method is controlled around 0.2 m.

## Introduction

Underwater robots have the capability to perform a wide range of tasks, eliminating the need for human intervention. These tasks include underwater biological observation, pipeline inspection, resource exploration, and underwater reconnaissance [1–4]. In nature, fish possess exceptional underwater locomotion abilities, allowing them to swim swiftly and efficiently [5]. By emulating the movement patterns of fish, bionic underwater robots exhibit superior mobility, efficiency, and speed compared to traditional propeller-driven underwater robots. These bionic robots also demonstrate improved adaptability to the complex underwater environment [6].

In recent years, substantial advancements have been made in the development of fish-like underwater robots. Simons et al. [7] introduced Galatea, a robotic fish propelled by both pectoral and caudal fins. This design allows for control over depth, movement patterns, and underwater orientation. Zhang et al. [8] presented a dual-caudal-fin robotic fish, which exhibits exceptional stability and speed, capable of reaching a maximum speed of 1.21 BL/s. Wang et al. [9–11] developed Robcutt, a third-generation robotic fish specifically designed for deep-sea inspection and other underwater tasks.

The autonomous avoidance of underwater obstacles and efficient trajectory planning are crucial for bionic underwater robots to successfully complete their underwater tasks. Extensive research has been conducted in this area by scholars. Sani et al. [12] developed a trajectory controller based on the proportional-integral-derivative (PID) method, which utilizes autonomous underwater vehicle (AUV) position, velocity, and acceleration data to plan trajectories. This method demonstrates high accuracy and robustness. Filaretov et al. [13] employed Bessel curves to generate smooth trajectories for AUVs in obstacle-free environments. Wang et al. [14] designed a real-time dynamic Dubins–Helix method for trajectory planning in obstacle-free environments, resulting in well-smoothed trajectories. Murthy et al. [15] proposed a spline curve-based trajectory planning method to address the terrain following problem for AUVs in obstacle-free environments. Zeng et al. [16] employed B-spline curves to rapidly plan optimal trajectories for underwater robots in static obstacle environments. Li et al. [17] combined cubic B-spline curves with genetic algorithms to design a trajectory planning method for underdriven underwater robots, effectively matching the kinematic capabilities of AUVs. Wang et al. [18] achieved collaborative optimal trajectory planning for multiple robots operating in static obstacle environments by utilizing temporal-spatial Bezier curves. Gan et al. [19] introduced a 2D trajectory planning model for underwater environments that utilizes the repulsive force field method. This approach demonstrates effective trajectory planning results, particularly in scenarios with a mix of dynamic and static obstacles. Reinforcement learning algorithms have enabled underwater robots to interact directly with the environment, offering improved planning effectiveness and adaptability in complex and ever-changing underwater conditions. Wang et al. [20] developed an online learning algorithm based on reinforcement learning, which plans optimal trajectories for AUVs in obstacle-free under-ice environments. Yang et al. [21] devised a value-based reinforcement learning algorithm that allows planned trajectories to adapt to varying oceanic conditions. Hadi et al. [22] designed a trajectory planning method based on deep reinforcement learning algorithms, enhancing the robustness and adaptability of trajectory planning in dynamic obstacle environments.

While the aforementioned trajectory planning methods have yielded positive results, they face challenges in complex and dynamic underwater environments where obstacles are prone to movement. This dynamic nature of underwater obstacles poses greater difficulty for underwater obstacle avoidance. Furthermore, there is a scarcity of studies focusing on trajectory planning for underwater environments with dynamic obstacles. Although reinforcement learning methods exhibit superior adaptability to the environment and perform well in multidynamic obstacle underwater scenarios, there is still room for improvement in terms of planning efficiency, trajectory length, and smoothness.

During the operation of bionic underwater robots, ensuring accurate trajectory tracking and reaching the desired destination is equally crucial, making motion control research of paramount importance. Wang et al. [23] successfully achieved path-point tracking control on a homemade cuttlefish-imitating underwater robot called Robcutt-II by combining a low-complexity guidance system with the backstepping method. Their approach demonstrated excellent accuracy and efficiency. Oh et al. [24] tackled the path-point tracking control problem by combining line-of-sight navigation principles with the model predictive control method. Wang et al. [25] designed a path tracking controller based on active disturbance rejection control, which exhibited higher accuracy and stability compared to traditional PID methods. Chen et al. [26] developed a path tracking controller utilizing a fuzzy control algorithm optimized by a genetic algorithm, resulting in improved accuracy and robustness compared to conventional fuzzy controllers. Zhang et al. [27] effectively integrated the deep deterministic policy gradient algorithm with an adaptive multiple constraint controller for both path tracking control and obstacle avoidance. Their approach demonstrated commendable algorithmic efficiency and stability. Wang et al. [28] designed a spline-bridged elite-duplication genetic algorithm–deep reinforcement learning framework by combining elite-duplication genetic algorithm with deep reinforcement learning. The integration of path planning, path smoothing, and path tracking control for unmanned surface vehicle (USV) in congested waters was realized. Wang et al. [29] designed a data-driven performance-prescribed reinforcement learning control scheme to achieve optimal control without the need of USV modeling. It has good trajectory tracking control accuracy and stability in a complex environment with multiple obstacles. Cui et al. [30] developed a trajectory tracking controller based on deep reinforcement learning algorithms, yielding favorable outcomes in terms of control accuracy and stability. Wang et al. [31] established a reinforcement learning-based optimal tracking control scheme. It has good accuracy and stability for the trajectory tracking control problem of USV in the presence of complex unknowns. Trajectory tracking enables an underwater robot to follow a desired path according to a specified time pattern. It is very important to enhance the motion accuracy of the robot and improve the efficiency of underwater work. However, the majority of existing studies on tracking control for bionic underwater robots primarily focus on path point tracking control and path tracking control. Trajectory tracking, which involves temporal-spatial synchronization, is a more complex task, leading to a limited number of studies in this particular area.

Therefore, this article focuses on the above issues for research. Efforts have been made to solve the trajectory planning problem in underwater multidynamic obstacle environments, and a trajectory planning method combining temporal-spatial Bezier curve and improved proximal policy optimization (IPPO) algorithm has been proposed. First, we plan a smooth global trajectory using temporal-spatial Bezier curves. Then, we use the IPPO algorithm to perform real-time local trajectory replanning during the bionic underwater robot’s traveling along the global trajectory. Dynamic obstacles on the global trajectory are avoided, while the bionic underwater robot quickly returns to the global trajectory. The performance of trajectory planning is improved while solving the trajectory planning problem of the bionic underwater robot in a multidynamic obstacle environment. Finally, a fuzzy PID controller is designed to control the bionic underwater robot to track the planned trajectory, and the performance of this article’s method is demonstrated through simulation and comparative experiments.

In the remainder of this paper, Materials and Methods describes the bionic underwater robot trajectory planning problem description and details the trajectory planning scheme. Results describes the simulation experiment design and experimental results. Finally, Discussion gives conclusions and outlook.

## Materials and Methods

### RoboDact system design

**Fig. 1.**
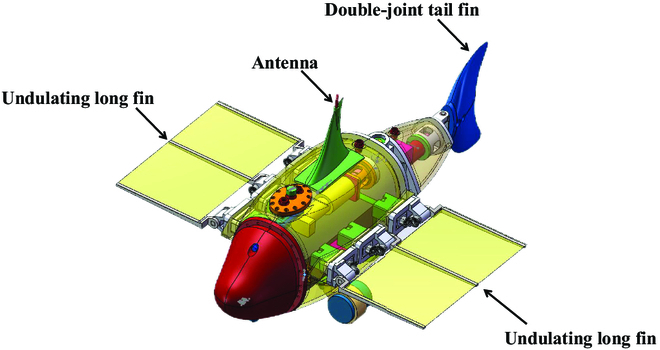
Schematic of the BUV mechanism.

This research article utilizes the bionic underwater robot, RoboDact, as an experimental platform to validate the proposed trajectory planning method. As shown in Fig. 1, RoboDact has a pair of wavy pectoral fins and a double-jointed caudal fin. It moves using a hybrid propulsion mode and can swim fast in broadly categorized body/caudal fin mode while having good stability in median/paired fin mode. Three identical servo motors are mounted on each side of the main control cabin to drive the undulatory pectoral fins for stabilized movement. A 200-W DC motor is mounted at the rear of the main control cabin to drive the lumbar joint, while a 90-W DC motor and driver are mounted in the caudal cabin to jointly drive the tail fin to oscillate for fast swimming. In addition, the DC motor driver, power management unit and servo motor driver are all installed in the main control cabin. Table 1 lists the relevant parameters of RoboDact [32].

**Table 1.** RoboDact measurement parameters

Parameters	Value
Main body length	706.5 mm
Main body width	248.0 mm
Main body height	240.0 mm
Maximum speed	61.12 cm/s
Total weight	15.1 kg
Total buoyancy	148.5 N

### RoboDact dynamics model

The trajectory planning problem addressed in this article pertains to 3-dimensional (3D) space. However, considering the characteristics of RoboDact, which includes a large metacentric height and excellent static stability, this study disregards its motionin the pitch and roll degrees of freedom. Moreover, given the relatively low-speed motion of bionic underwater robots, this research simplifies the analysis by assuming linear damping and disregarding nonlinear damping effects [33]. As a result, the kinematic model of RoboDact can be represented as follows:

$$\dot{\eta }=J\left(\psi \right)\nu$$ (1)

where $\dot{\eta }={\left[x,\;y,\;z,\;\psi \right]}^{T}$ is the position and heading of the bionic underwater robot in the world coordinate system. *ν* = [*u*, *v*, *w*, *r*]*^T^* is the velocity of the bionic underwater robot in the follower coordinate system. *J* is the rotational transformation matrix, which can be expressed as:

$$J=\left[\begin{array}{cccc} \cos\left(\psi \right) & -\sin\left(\psi \right) & 0 & 0\\ \sin\left(\psi \right) & \cos\left(\psi \right) & 0 & 0\\ 0 & 0 & 1 & 0\\ 0 & 0 & 0 & 1 \end{array}\right]$$ (2)

The dynamics model of RoboDact can be represented as follows:

$$M\dot{\nu }=-\;C\left(\nu \right)\nu -D\nu +\tau +\tau_{d}$$ (3)

where *M* is the mass and additional mass matrix. *D* is the linear damping matrix. *C* is the Coriolis force and centripetal force matrix. *τ* = [*τ_u_*, 0, *τ_w_*, *τ_r_*] describes body-fixed thrust and torque (on surge, heave, and yaw). *τ_d_* = [*τ_du_*, *τ_dv_*, *τ_dw_*, *τ_dr_*] represents the disturbance force and torque (on surge, sway, heave, and yaw). Since RoboDact has a bilateral symmetric structure, the matrices *M* and D can be expressed as:

$$M=\left[\begin{array}{cccc} m_{11} & 0 & 0 & 0\\ 0 & m_{22} & 0 & m_{24}\\ 0 & 0 & m_{33} & 0\\ 0 & m_{24} & 0 & m_{44} \end{array}\right],D=\left[\begin{array}{cccc} d_{11} & 0 & 0 & 0\\ 0 & d_{22} & 0 & d_{24}\\ 0 & 0 & d_{33} & 0\\ 0 & d_{24} & 0 & d_{44} \end{array}\right]$$ (4)

Meanwhile *C* can be expressed as:

$$C\left(\nu \right)=\left[\begin{array}{cccc} 0 & 0 & 0 & -m_{22}\upsilon -m_{24}r\\ 0 & 0 & 0 & m_{11}u\\ 0 & 0 & 0 & 0\\ m_{22}\upsilon +m_{24}r & -m_{11}u & 0 & 0 \end{array}\right]$$ (5)

The actual amount of control over the bionic underwater robot is actually the parameters of the waves in its pair of pectoral and one caudal fins, including frequency *f*, amplitude *A*, and deflection angle *θ*. The following equation was used to fit the thrust or torque to the above control parameters on the actual RoboDact machine:

$$\left\{\begin{array}{c} \tau_{u}=k_{u1}\tanh\left(k_{u2}A\right)\left(f_{L}+f_{R}\right)+k_{u3}\tanh\left(k_{u4}A_{\text{Tail}}\right)f_{\text{Tail}}\\ \tau_{\omega }=k_{\omega 1}\tanh\left(k_{\omega 2}A\right)\tanh\left(k_{\omega 3}\theta \right)\left(f_{L}+f_{R}\right)\\ \tau_{r}=k_{r1}\tanh\left(k_{r2}A\right)\left(f_{L}-f_{R}\right) \end{array}\right.$$ (6)

where *k*_*u*1_, *k*_*u*2_, *k*_*u*3_, *k*_*u*4_, *k*_*ω*1_, *k*_*ω*2_, *k*_*ω*3_, *k*_*r*1_, and *k*_*r*2_ are some characteristic parameters. Equation 6 is derived from a series of force measurements and motion experiments [34,35].

### Trajectory planning problem description

**Fig. 2.**
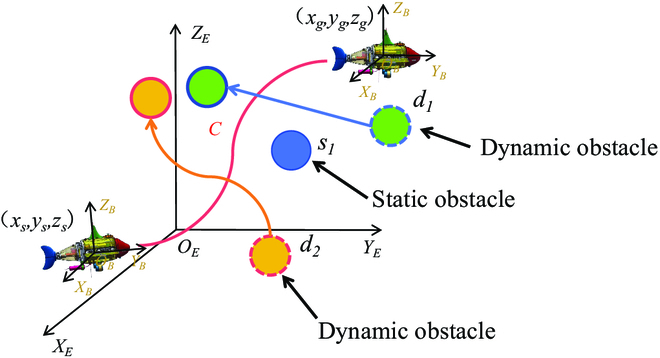
Schematic of the trajectory planning problem.

The trajectory planning problem can be defined as follows: Given the physical limitations and global obstacles present in the environment, the objective is to devise a feasible trajectory that allows the bionic underwater robot to navigate from its initial position to the desired end position. Additionally, in the event of encountering local dynamic obstacles during the robot’s traversal, the proposed approach enables real-time trajectory replanning to ensure obstacle avoidance and uninterrupted progress. As shown in Fig. 2, the initial position of RoboDact is (*x_s_*, *y_s_*, *z_s_*) in the world coordinate system *O_E_X_E_Y_E_Z_E_*. The target position is (*x_g_*,* y_g_*, *z_g_*) in the world coordinate system. *O_B_X_B_Y_B_Z_B_* is the follower coordinate system. *C* is the planned feasible trajectory. *s_i_* and *d_i_* is multiple static obstacles and dynamic obstacles in the environment, respectively. Then, the trajectory planning problem can be expressed as:

$$F_{s}(x_{s},y_{s},z_{s},{\mathrm{vx}}_{s},{\mathrm{vy}}_{s},{\mathrm{vz}}_{s})\overset{C}{\to }\;F_{g}(x_{g},y_{g},z_{g},{\mathrm{vx}}_{g},{\mathrm{vy}}_{g},{\mathrm{vz}}_{g})$$ (7)

where *F_s_* is the initial state of the bionic underwater robot. *F_g_* is the target state of the bionic underwater robot. (*vx_s_*, *vy_s_*, *vz_s_*) is the initial velocity of the bionic underwater robot. (*vx_g_*, *vy_g_*, *vz_g_*) is the target velocity of the bionic underwater robot.

### Real-time dynamic trajectory planning

**Fig. 3.**
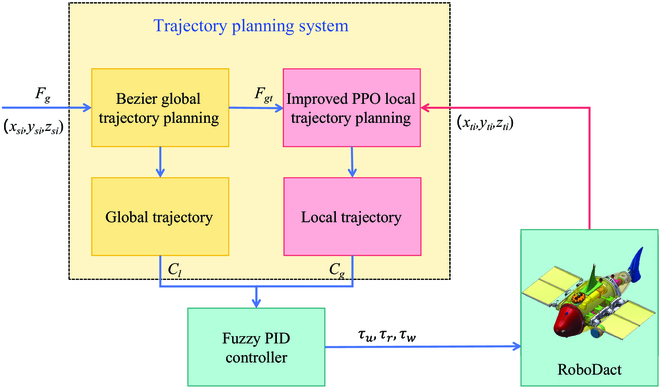
The block diagram of the trajectory planning system.

This section proposes a solution for the trajectory planning problem of the bionic underwater robot RoboDact. As shown in Fig. 3, the trajectory planning scheme mainly consists of a global trajectory planning part, a local trajectory planning part, and a motion controller. The first part plans a smooth global trajectory from the start point to the end point for RoboDact. The initial positions (*x_si_*, *y_si_*, *z_si_*) of all dynamic obstacles *i* are input with the target state *F_g_*, and the Bezier curve gives a smooth global trajectory *C_g_* based on the input. The second part is the local trajectory planning part. All obstacles in the environment move dynamically, and when RoboDact moves along the global trajectory *C_g_*, there may be obstacles moving to the global trajectory. This requires an improved PPO algorithm for real-time local trajectory replanning. The start position is set to the current position of the bionic underwater robot (*x_st_*, *y_st_*, *z_st_*), and the target position is set to the appropriate position on the global trajectory *C_g_* that crosses the obstacles (*x_gt_*, *y_gt_*, *z_gt_*). The real-time states of all obstacles (*x_ti_*, *y_ti_*, *z_ti_*, *v_ti_*) are input into the trained model to give a local trajectory *C_l_* that brings the RoboDact back to the global trajectory *C_g_*. The third part is the motion controller, which is used to control the motion of the bionic underwater robot to follow the planned trajectory *C* to the target position. The actual control inputs to the RoboDact are the parameters of the 3 fin-on-fin waves that include frequency *f*, amplitude *A* and offset angle *θ*.

#### Global trajectory planning section

In this section, we use temporal-spatial Bezier curves to provide global trajectory planning for RoboDact. We use cubic Bezier curves, which have 4 control points *P*_0_, *P*_1_, *P*_2_, and *P*_3_, and the cubic Bezier curves can be expressed as:

$$P\left(\tau \right)=\sum_{i=0}^{3}\;B_{i,3}\left(\tau \right)P_{i}$$ (8)

$$B_{i,3}\left(\tau \right)=C_{3}^{i}{\left(1-\tau \right)}^{3-i}\tau^{i}$$ (9)

where *B*_*i*,3_(*τ*) is called the Bernstein basis function. *P_i_* are the coordinates of the *i*th control point.

The cubic Bezier curves has 2 properties:

• Endpoint property: For cubic Bezier curves, there are at *τ* = 0 and *τ* = 3, respectively:

$$P\left(0\right)=P_{0}$$ (10)

$$P\left(1\right)=P_{3}$$ (11)

• Cut vector property: Derivation of the above equation gives the direction of the tangent lines to the 2 end points at the start and end of the Bezier curve respectively:

$$P^{\prime }\left(0\right)=3\left(P_{1}-P_{0}\right)$$ (12)

$$P^{\prime }\left(1\right)=3\left(P_{3}-P_{2}\right)$$ (13)

From the Bezier curve mathematical expression, when we introduce the time axis in the Bezier curve, we can correspond the cubic Bezier curve to the 4-dimensional space-time, which can be expressed as:

$$P\left(\tau \right)=B\left(\tau \right)\left[\begin{array}{cccc} x_{0} & y_{0} & z_{0} & t_{0}\\ x_{1} & y_{1} & z_{1} & t_{1}\\ x_{2} & y_{2} & z_{2} & t_{2}\\ x_{3} & y_{3} & z_{3} & t_{3} \end{array}\right]$$ (14)

where (*x_i_*, *y_i_*, *z_i_*, *t_i_*) is the spatiotemporal position of the control point *P_i_*. By the 2 properties of the Bezier curve, only the temporal-spatial positions of points *P*_0_ and *P*_3_ need to be known to determine the Bezier curve. At *τ* = 0 and *τ* = 1, it can be obtained:

$$\left\{\begin{array}{cc} x_{1}=x_{0}+v_{x0}\left(t_{1}-t_{0}\right) & \\ y_{1}=y_{0}+v_{y0}\left(t_{1}-t_{0}\right) & \\ z_{1}=z_{0}+v_{z0}\left(t_{1}-t_{0}\right) & \end{array}\right.,\left\{\begin{array}{cc} x_{2}=x_{3}+v_{x3}\left(t_{3}-t_{2}\right) & \\ y_{2}=y_{3}+v_{y3}\left(t_{3}-t_{2}\right) & \\ z_{2}=z_{3}+v_{z3}\left(t_{3}-t_{2}\right) & \end{array}\right.$$ (15)

That is to say, it is only necessary to determine the moments *t_i_* of the 4 control points to determine the spatial positions of *P*_1_ and *P*_2_. In addition, *t*_0_ and *t*_3_ are known, so the global trajectory planning problem for Bezier curves can be transformed into an optimization problem for 2 time parameters *t*_1_ and *t*_2_. We use the particle swarm optimization algorithm to solve the optimization problem. The optimization objective is the shortest total distance *S* of the trajectory, and *S* can be expressed by accumulating each small distance:

$$S=\sum_{△L}\;\sqrt{{\left(△x\left(\tau \right)\right)}^{2}+{\left(△y\left(\tau \right)\right)}^{2}+{\left(△z\left(\tau \right)\right)}^{2}}$$ (16)

Considering the various constraints of the trajectory planning problem, the objective function expression is defined as follows:

$$f\left(t_{1},t_{2}\right)=S+\sum M_{k}\cdot \delta_{k}$$ (17)

where the parameter *M_k_* is a very large number. *δ_k_* is the various constraints of the trajectory planning problem, including velocity constraints, acceleration constraints, curvature constraints, obstacle avoidance constraints, and so on.

Thus, the optimization problem can be expressed as:

$$\begin{array}{c} \underset{t_{1},t_{2}}{\text{min}}\;f\left(t_{1},t_{2}\right)\\ s.t.\;t_{0}\leq t_{1},t_{2}\leq t_{3} \end{array}$$ (18)

The initial number of particles is set to *M*. The dimension is 2 corresponding to the 2 time parameters *t*_1_ and *t*_2_, and the maximum number of iterations is *T*. According to the expression:

$$v_{j,k+1}=c_{0}v_{j,k}+c_{1}\left(p_{j,k}-x_{j,k}\right)+c_{2}\left(g_{k}-x_{j,k}\right)$$ (19)

$$x_{j,k+1}=x_{j,k}+v_{j,k}$$ (20)

where *v*_*j*,*k*_, *x*_*j*,*k*_ are the position and velocity of the *j*th particle at the *k*th iteration, respectively. *p*_*j*,*k*_ is the optimal solution position of the *j*th particle in the previous *k* iterations. *g_k_* is the optimal solution position of the whole particle swarm in the previous *k* iterations. *c*_0_, *c*_1,_ and *c*_2_ are the population cognition coefficients. After *T* iterations, the shortest distance can be finally obtained.

#### Local trajectory planning section

**Fig. 4.**
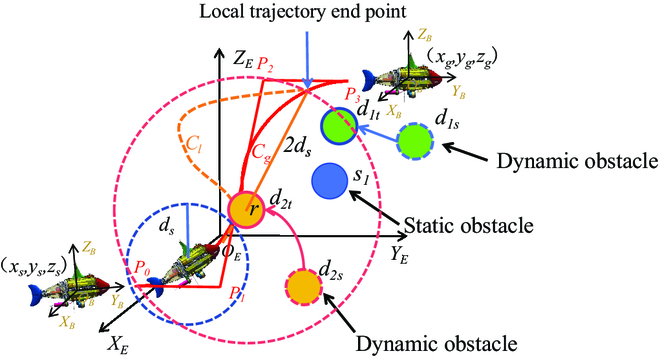
Schematic of the local trajectory planning problem.

In this section, the improved PPO algorithm is used to provide local trajectory planning for RoboDact. As shown in Fig. 4, when the bionic underwater robot advances along the global trajectory and detects that there is a dynamic obstacle moving to the global trajectory within a safe distance *d_s_* in front of it, it needs to carry out real-time local trajectory replanning through the improved PPO algorithm to get back to the global trajectory bypassing the obstacle. The obstacles in the environment are spherical obstacles with a radius *r* from the center of the sphere as their surface location. The starting point of local trajectory planning is set as the current position (*x_st_*, *y_st_*, *z_st_*) of the bionic underwater robot. Since the target position is set too close to the starting point may affect the effectiveness of local trajectory planning, the point at 2*d_s_* from the obstacle surface of the global trajectory is taken as the target position (*x_gt_*, *y_gt_*, *z_gt_*). The trained strategy model is utilized to make the bionic underwater robot quickly return to the global trajectory around the obstacle. Note that when the bionic underwater robot is about to reach the target position and detects an obstacle moving to the target position. The target position is updated to a point 2*d_s_* from the surface of the new obstacle and local trajectory planning continues. During the local trajectory planning process, the bionic underwater robot acts directly with the environment. The state *s*_*t*+1_ of the bionic underwater robot at the next moment depends only on the state *s_t_* at the previous moment.

Design the IPPO algorithm state as:

$$s={\left[d_{i,obs},\;v_{i,obs},\;d_{goal}\right]}^{T}$$ (21)

where *d*_*i*,*obs*_, *v*_*i*,*obs*_ are the distance and speed of the bionic underwater robot from all obstacles within the current safe distance, respectively. *d_goal_* is the distance of the bionic underwater robot from the target location.

Action *a* is designed as the position information of the next moment of the bionic underwater robot, denoted as:

$$a={\left[\rho ,\;\sigma ,\;\theta \right]}^{T}$$ (22)

where *ρ*,*σ*,*θ* is the angle from the current position of the bionic underwater robot to the next position in the world coordinate system with the 3D coordinate axis, according to which the next moment state *s* of the bionic underwater robot can be updated.

Since the real-time local trajectory planning process, the bionic underwater robot needs to avoid all dynamic obstacles at each moment while quickly reaching the target position and returning to the global trajectory. Therefore, the reward function needs to take both tasks into account and is designed as:

$$\left\{\begin{array}{cc} \frac{{\mathrm{dis}}_{\mathrm{obs}}-r}{r}-1 & \text{if}\;{\mathrm{dis}}_{\mathrm{obs}}<r\\ \frac{{\mathrm{dis}}_{\mathrm{obs}}-r-0.9}{r+0.9}-0.5 & \text{if}\;r<{\mathrm{dis}}_{\mathrm{obs}}<r+0.9\\ \frac{-{\mathrm{dis}}_{\text{goal}}}{{\mathrm{dis}}_{\text{sgoal}}} & \;\text{if}\;\text{not}\;\text{goal}\\ \frac{-{\mathrm{dis}}_{\text{goal}}}{{\mathrm{dis}}_{\text{sgoal}}}+3 & \text{if}\;\text{goal} \end{array}\right.$$ (23)

where *dis_obs_* is the distance between the bionic underwater robot and the center of the nearest obstacle. *r* is the radius of the obstacle. *r+0.9* is the safe distance between the bionic underwater robot and the surface of the obstacle. The first 2 items of the reward function cause the bionic underwater robot to avoid obstacles by applying varying degrees of punishment and to maintain a safe distance from the obstacles as much as possible. *dis_goal_* is the distance between the bionic underwater robot and the end point. *dis_sgoal_* is the distance between the start and the end point. The latter 2 items make the bionic underwater robot converge to the end point through reward and punishment. The reward and penalty coefficients in the reward function are obtained after several adjustments by observing the training effect.

IPPO algorithm is a reinforcement learning algorithm based on Actor-Critic network with good stability and convergence speed, where the Actor network input dimension is the state dimension state_dim. The output dimension is action_dim, denoting the average Gaussian decision value for each action. The Critic network input dimension is the state dimension and the output dimension is the reward value *V* of the evaluated state, denoted as:

$$V_{\pi }\left(s\right)=E_{\pi }\left[\sum_{k=0}^{\infty }\;\gamma^{k}R_{t+k+1}\left|S_{t}=s\right.\right]$$ (24)

IPPO algorithm is a strategy gradient improvement algorithm; the core operation is the loss function. The IPPO algorithm improves the loss function to improve the strategy gradient sensitive to the learning rate. The loss function can be expressed as:

$$L^{\mathrm{CPI}}\left(\theta \right)=\hat{E_{t}}\left[\frac{\pi_{\theta }\left(a_{t}\left|s_{t}\right.\right)}{\pi_{\text{θold}}\left(a_{t}\left|s_{t}\right.\right)}\hat{A_{t}}\right]=\hat{E_{t}}\left[r_{t}\left(\theta \right)\hat{A_{t}}\right]$$ (25)

where *π_θ_*(*a_t_*|*s_t_*) and *π_θold_*(*a_t_*|*s_t_*) are the new and old strategies, respectively. $\hat{A_{t}}$ is the dominance function.

When the new update parameters differ too much or too little from the old network, the policy network will be over-updated and fall into a local optimum, which is not conducive to convergence. Therefore, IPPO introduces the CLIP function for limitation, and the loss function is finally expressed as:

$$L^{\text{CLIP}}\left(\theta \right)=\hat{E_{t}}\left[\min\left(r_{t}\left(\theta \right)\hat{A_{t}},\text{clip}\left(r_{t}\left(\theta \right),1-\epsilon ,1+\epsilon \right)\hat{A_{t}}\right)\right]$$ (26)

where ϵ is the clip parameter. The dominance function $\hat{A_{t}}$ can be expressed as:

$$A^{\pi }\left(s_{t},\;a_{t}\right)=Q^{\pi }\left(s_{t},\;a_{t}\right)-V^{\pi }\left(s_{t}\right)$$ (27)

where *V^π^*(*s_t_*) is the expected value of rewards for all actions in state *s_t_*, which is the weighted average value. *Q^π^*(*s_t_*, *a_t_*) is the reward value obtained by performing action *a_t_* in state *s_t_*.Therefore, the model can be trained using this loss function.

#### Motion controller

In this section, a fuzzy PID controller is used as the motion controller of RoboDact. After the trajectory planning is completed, RoboDact receives the control signal to track the planned trajectory through the motion controller. The fuzzy PID controller is based on the PID algorithm. The parameters of the PID controller *K_p_*, *K_i_*, *K_d_* are adjusted in real time by the fuzzy control method, so that the motion controller has a certain degree of adaptive ability. The controller can be expressed as:

$$\left\{\begin{array}{cc} e\left(k\right)=x_{d}-x_{c}\\ \mathrm{ec}\left(k\right)=e\left(k\right)-e\left(k-1\right) & \end{array}\right.$$ (28)

where *e*(*k*) is the input error. *ec*(*k*) is the derivative of the input error. *x_c_* is the current position of the machine fish, and *x_d_* is the desired position.

The PID controller law can be expressed by the following equation:

$$\tau =K_{p}\cdot e+K_{i}\cdot \int e\cdot \mathrm{dt}+K_{d}\cdot \dot{e}$$ (29)

where *K_p_*, *K_i_*, *K_d_* are the proportional, derivative and integral gains of the PID controller respectively. All the 3 parameters are calculated by the fuzzy controller which uses Mamdani type for adaptive optimization of the gains.

The fuzzy controller is generated with reference to the following 3 key principles. The generation principles refer to [36]:

• The proportional gain *K_p_* has the effect of reducing the rise time and steady-state error. Therefore, when the input error *e* is large, the *K_p_* value should be increased appropriately to improve the response speed of the machine fish. When *e* is medium in size, take a smaller value of *K_p_* so that the system has a smaller amount of overshoot to ensure the response speed. When *e* is small, the *K_p_* value is adjusted to a larger value to reduce the static difference and improve the control accuracy. Therefore, the fuzzy rule control table for *K_p_* is shown in the Table 2.
• The integral gain *K_i_* has the effect of eliminating static errors. When the input error *e* is large, *K_i_* should be small to prevent integral saturation. When *e* is medium in size, *K_i_* should be relatively moderate to avoid affecting stability. When *e* is small, *K_i_* should be increased to reduce the regulation static difference. Therefore, the fuzzy rule control table for *K_i_* is shown in the Table 3.
• The derivative gain *K_d_* has the effect of smoothing the dynamics of the system. When the input error *e* is large, *K_d_* should be increased to obtain a smaller overshoot. When *e* is medium in size, *K_d_* should be appropriately small and kept constant. When *e* is small, *K_d_* should be small to minimize the controlled process. Therefore, the fuzzy rule control table for *K_d_* is shown in the Table 4.

**Table 2.** Fuzzy control rules for *K_p_*

*K_p_*	NB	NM	NS	ZO	PS	PM	PB
NB	PB	PB	PM	PM	PS	ZO	ZO
NM	PB	PB	PM	PS	PS	ZO	NS
NS	PM	PM	PM	PS	ZO	NS	NS
ZO	PM	PM	PS	ZO	NS	NM	NM
PS	PS	PS	ZO	NS	NS	NM	NM
PM	PS	ZO	NS	NM	NM	NM	NB
PB	ZO	ZO	NM	NM	NM	NB	NB

**Table 3.** Fuzzy control rules for *K_i_*

*K_i_*	NB	NM	NS	ZO	PS	PM	PB
NB	NB	NB	NM	NM	NS	ZO	ZO
NM	NB	NB	NM	NS	NS	ZO	NO
NS	NB	NM	NS	NS	ZO	PS	PS
ZO	NM	NM	NS	ZO	PS	PM	PM
PS	NM	NS	ZO	PS	PS	PM	PB
PM	ZO	ZO	PS	PS	PM	PB	PB
PB	ZO	ZO	PS	PM	PM	PB	PB

**Table 4.** Fuzzy control rules for *K_d_*

*K_d_*	NB	NM	NS	ZO	PS	PM	PB
NB	PS	NS	NB	NB	NB	NM	PS
NM	PS	NS	NB	NM	NM	NS	ZO
NS	ZO	NS	NM	NM	NS	NS	ZO
ZO	ZO	NS	NS	NS	NS	NS	ZO
PS	ZO	ZO	ZO	ZO	ZO	ZO	ZO
PM	PB	NS	PS	PS	PS	PS	PB
PB	PB	PM	PM	PM	PS	PS	PB

## Results

This section begins by verifying the effectiveness of the trajectory planning method proposed in this research paper within complex environments containing 5 dynamic obstacles. Through comparative experiments with traditional methods such as A* and rapidly exploring random tree (RRT) algorithms, we demonstrate the superiority of our approach in addressing trajectory planning problems. Additionally, we conduct semiphysical simulation experiments using the UWSim platform. Prior to these experiments, it is essential to train the local trajectory planning IPPO algorithm. During training, various parameters such as the starting point, end point, and dynamic obstacles within the environment are randomly simulated. Subsequently, the IPPO algorithm utilizes the data obtained from the interaction between the bionic underwater robot and the simulated environment to iteratively update the weights of the neural network until convergence is achieved, resulting in the acquisition of a trained strategy model.

### Trajectory planning simulation and comparative analysis

In this section, we first validate the planning effect of the trajectory planning method in a complex environment with 5 dynamic obstacles. The initial and end positions of the bionic underwater vehicle (BUV) are (0,2,5) and (10,10,5.5) in meters. Five dynamic obstacles with known initial positions are also set up, which are all moving with a certain function. The information of the given obstacles is as follows, and the length unit is meter in Tables 5 and 6.

**Table 5.** Obstacle information

Number	Initial position (m)	Radius (m)
1	(5,5,5)	1
2	(9,9,5.5)	1
3	(5,10,5.5)	1
4	(6,6,5)	1
5	(5,8,5)	1

**Table 6.** Obstacle information

Locomotor rhythm (m)	Movement speed (m/s)
(*x*_*t*−1_+*2cost*,*y*_*t*−1_+*2sint*,*z*_0_)	(*-0.5sint*,*0.5cost*,0)
(*x*_*t*−1_-*0.5t*,*y*_*t*−1_-*0.5t*,*z*_*t*−1_+*sin2t*)	(*-0.5*,*-0.5*,*0.5cos2t*)
(*x*_0_,*y*_*t*−1_-*t*,*z*_*t*−1_+*sin2t*)	(*0*,*-0.5*,*0.5cos2t*)
(*x*_*t*−1_+*3cos0.5t*,*y*_*t*−1_+*3sin0.5t*,*z*_*t*−1_+*sin2t*)	(*-0.5sin0.5t*,*0.5*,*0.5cos2t*)
(*x*_*t*−1_+*3sin0.5t*,*y*_*t*−1_+*3cos0.5t*,*z*_*t*−1_+*sin0.5t*)	(*-0.5cos0.5t*,*0.5*,*0.5sin2t*)

**Fig. 5.**
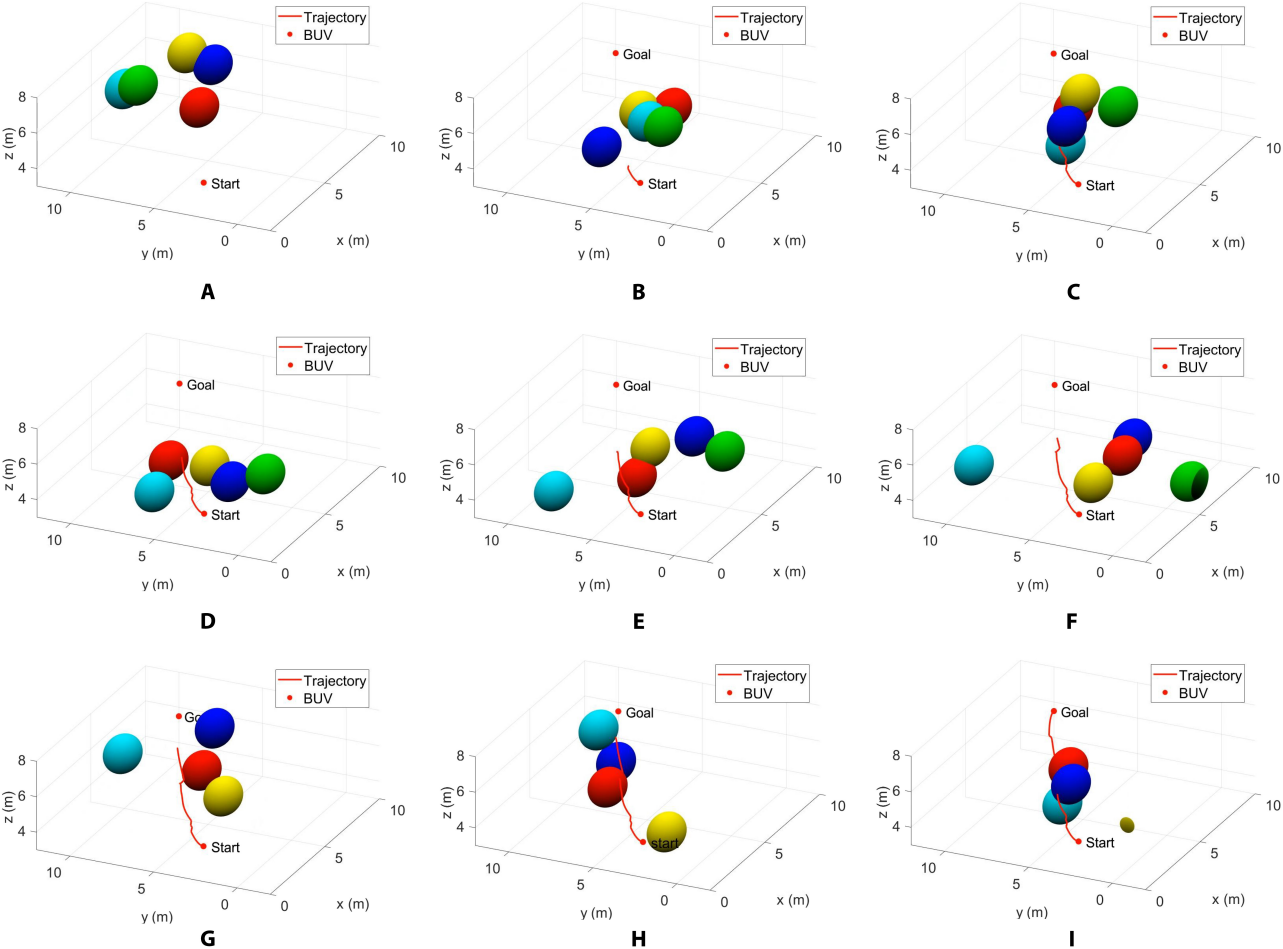
Three-dimensional space real-time trajectory planning results for BUV under 5-obstacles environment.

We get the results shown in Fig. 5 by numerical simulation in MATLAB. As can be seen from the figure, the motion process of the bionic underwater robot is divided into 3 stages:

Phase 1: From Fig. 5A to Fig. 5B, the BUV performs global trajectory planning based on the initial positions of all obstacles and moves along the Bezier curve. By the moment of Fig. 5B, the BUV detects the presence of obstacles within a safe distance on the global trajectory, so it starts local trajectory planning to avoid obstacles.

Phase 2: From Fig. 5B to Fig. 5G, the BUV performs local trajectory planning, which determines the starting point and target position of the local trajectory planning. Bypassing the obstacles, it reaches the target position at the moment of Fig. 5G and returns to the Bezier curve.

Phase 3: From Fig. 5G to Fig. 5I, the BUV continues along the global trajectory. It reaches the end point at the moment of Fig. 5I.

From Fig. 5, it can be seen that the method in this paper can well plan a trajectory that bypasses all the obstacles in a complex environment with 5 dynamic obstacles. Also, from Fig. 5F, the BUV can quickly move forward to the target location and return to the global trajectory after bypassing the obstacles by local trajectory planning.

**Fig. 6.**
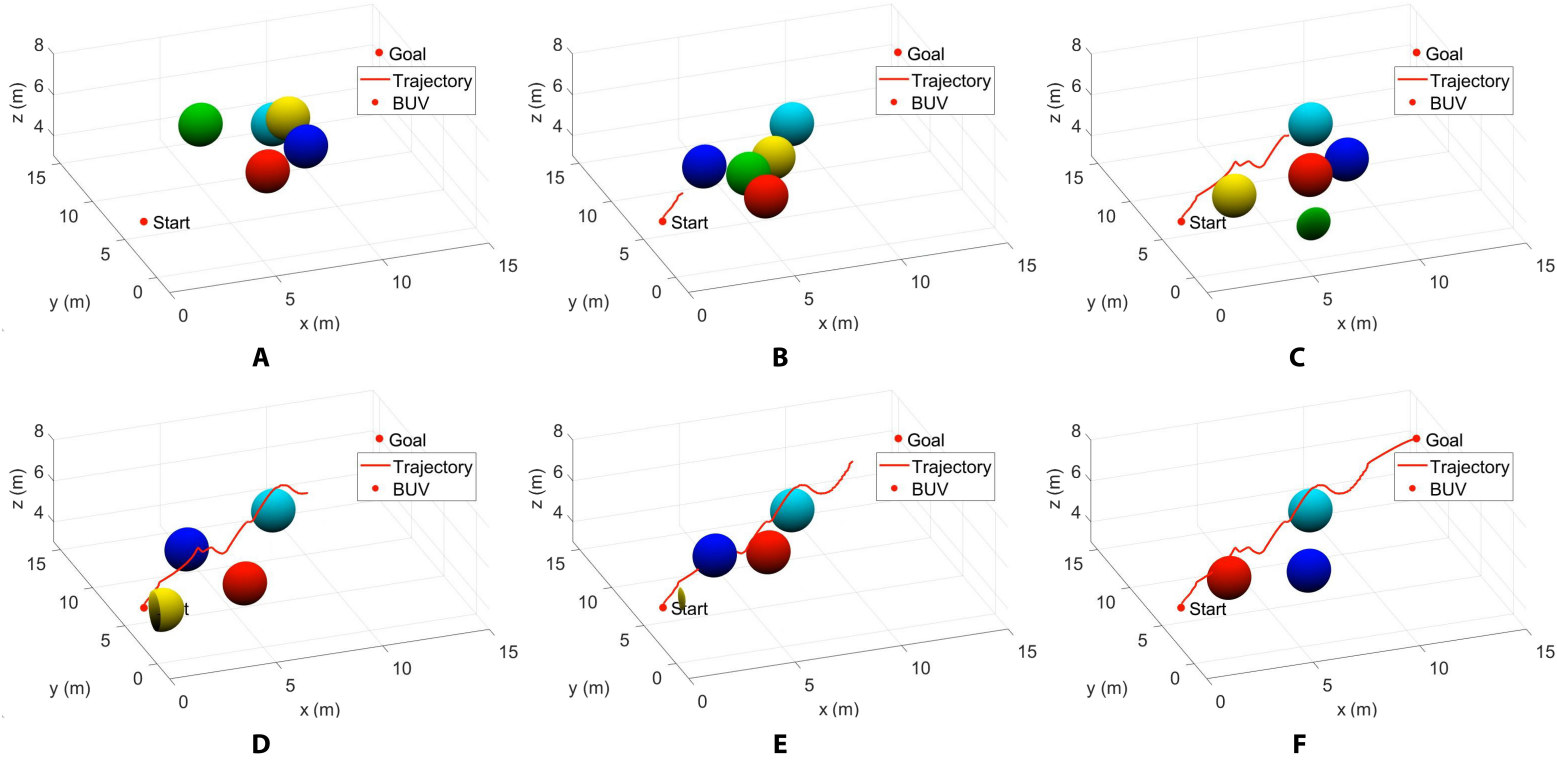
BUV 3-dimensional space real-time trajectory planning results for the case of local end point change.

Based on that experiment, we set obstacle 5 as an unknown stationary obstacle at the initial position. Its coordinates are set to (8.5,10,5.5) so that it is just at the end position of the first real-time local trajectory replanning. The information of the remaining 4 obstacles is consistent with Table 6. The initial and end positions of the BUV are (0,2,5) and (15,15,6). We get the results shown in Fig. 6 by numerical simulation in MATLAB. As can be seen from the figure, the motion process of the bionic underwater robot is divided into 4 stages:

Phase 1: From Fig. 6A to Fig. 6B, the BUV performs global trajectory planning based on the initial positions of all obstacles and moves along the Bezier curve. By the moment of Fig. 6B, the BUV detects the presence of obstacles within a safe distance on the global trajectory, so it starts local trajectory planning to avoid obstacles.

Phase 2: From Fig. 6B to Fig. 6C, the BUV performs local trajectory planning, determines the starting point and target location of the local trajectory planning and bypasses the obstacle. The obstacle 5 located at the end point of the local trajectory is detected at the moment of Fig. 6C, so the end point of the local trajectory planning is redetermined and the local trajectory replanning is performed with the current position as the starting point.

Phase 3: From Fig. 6C to Fig. 6E, the BUV performs localized trajectory planning to move toward the new end point and bypass the obstacles. It reaches the target position at the moment of Fig. 6E and returns to the Bezier curve.

Phase 4: From Fig. 6E to Fig. 6F, the BUV continues along the global trajectory. It reaches the end point at the moment of Fig. 6F.

Our method is compared and experimented with A* and RRT, which are traditional trajectory planning methods. In addition, the global trajectory planning using the reinforcement learning algorithm PPO is compared with the method in this paper. Trajectory planning isperformed for BUVs in an environment with multiple dynamic obstacles under the same initial conditions. Different algorithms were compared in terms of planning time, planning path length, and trajectory smoothness, respectively. The robot start position and end position are the same as above, and the obstacle information in the environment is also the same as Table 2.

**Fig. 7.**
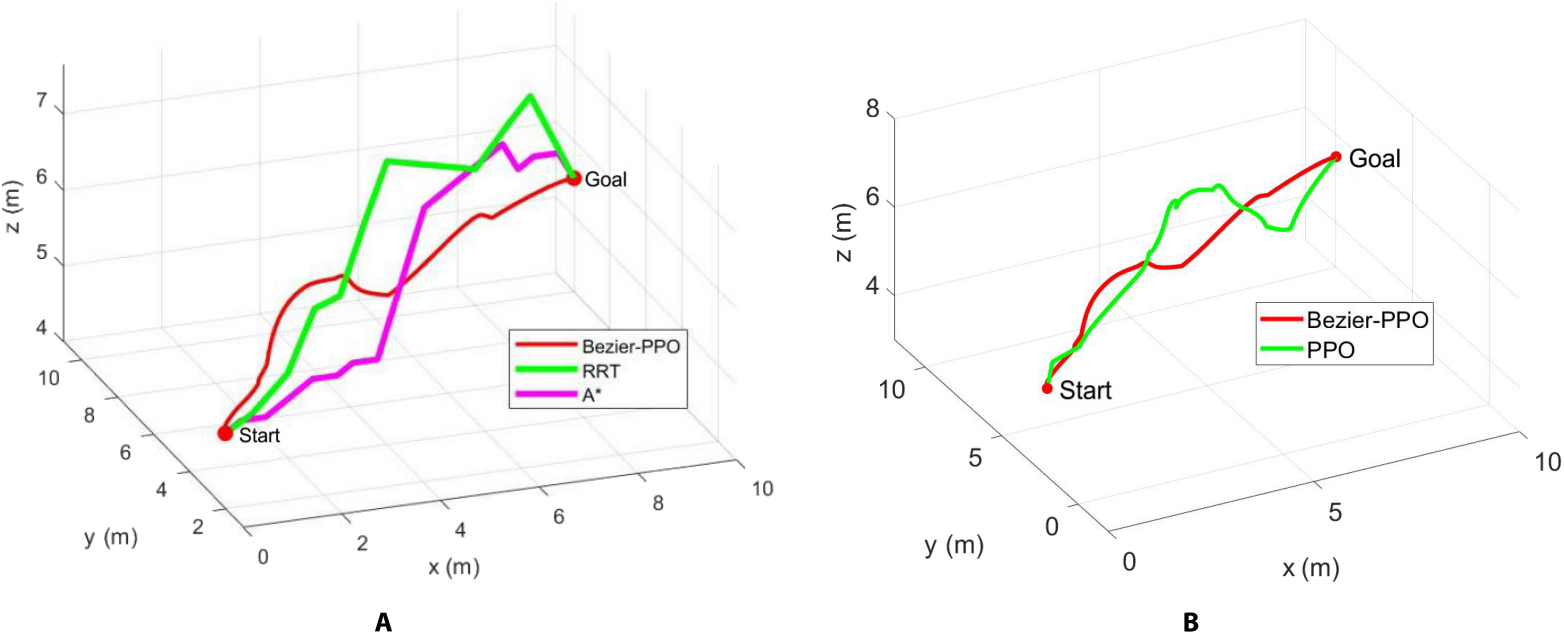
Trajectory comparison of 4 algorithms for a single BUV. (A) Comparison of Bezier-PPO, RRT, and A*. (B) Comparison of Bezier-PPO and PPO.

The comparison results are shown in Fig. 7. Table 7 gives the comparison results of the 4 algorithms in terms of planning time and distance traveled, where distance is in meters and time is in seconds.

**Table 7.** Comparison results

	Bezier-IPPO	A*	RRT	PPO
Planning time (s)	12.5	35.2	9.2	8.9
Total distance (m)	13.42	14.11	15.23	14.72

Our method is better than the A* algorithm in planning time, planning path length, and trajectory smoothness. In addition, our method is significantly better in trajectory length and smoothness, although it is not as good as RRT algorithm in planning time. Due to its algorithmic principle, the RRT algorithm produces different trajectories each time, making it difficult to obtain good trajectory curves. The better result of RRT in multiple simulations is given in Fig. 7. Compared with the PPO algorithm, the method in this paper utilizes Bezier curves in conjunction with the PPO algorithm, which is significantly better in terms of trajectory length and trajectory smoothness, although it has a little more planning time. Therefore, the method in this paper has certain advantages in the multidynamic obstacle trajectory planning problem.

### UWSim semiphysical simulation

**Fig. 8.**
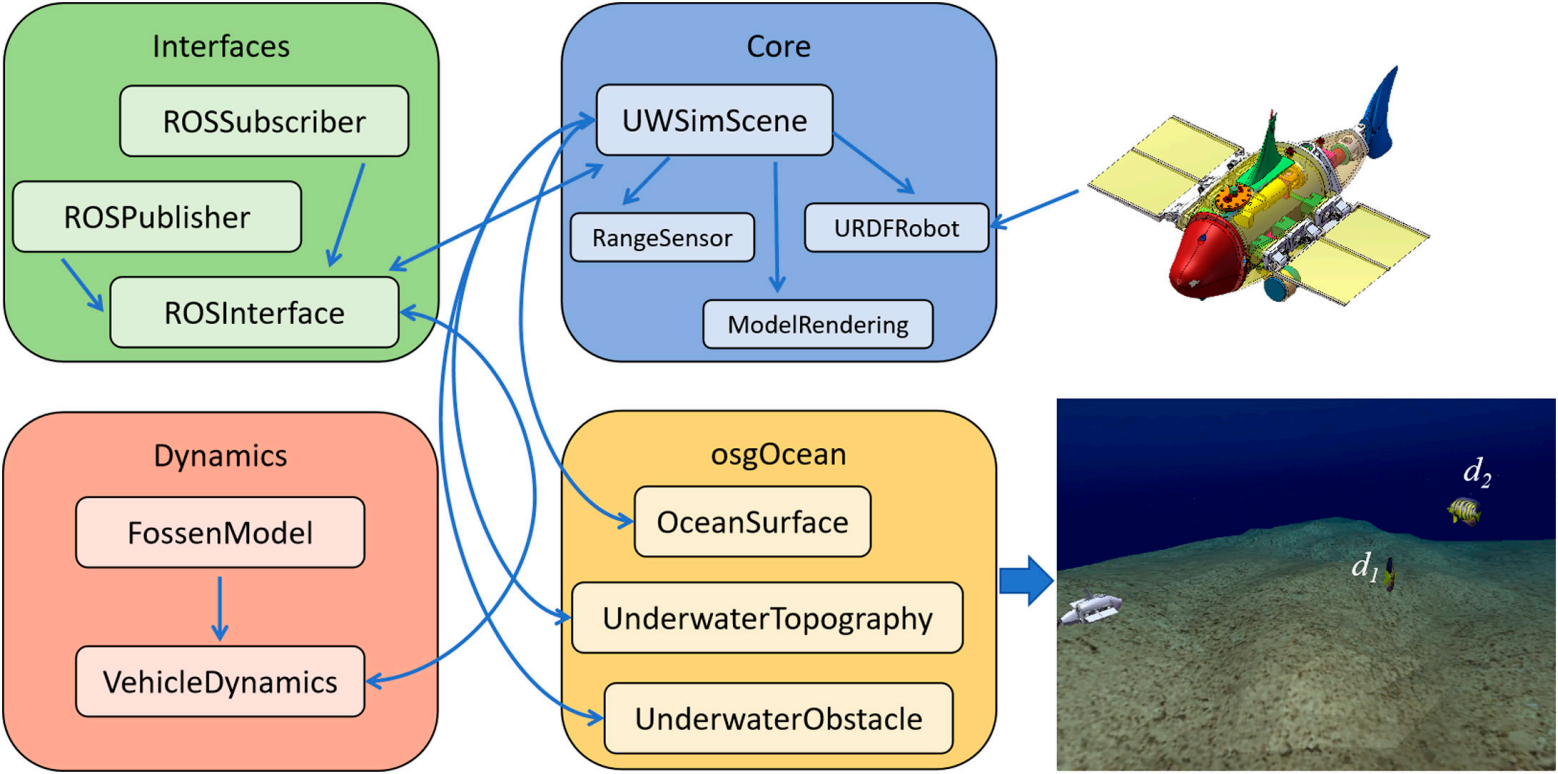
The block diagram of UWSim simulation platform.

This section presents the simulation experimental results of the proposed method using the UWSim platform. UWSim is a simulation platform for underwater robots that is based on ROS. It facilitates the loading of underwater robot models and provides an environment for testing integrated perception and control algorithms on robots [37]. As shown in Fig. 8, the UWSim simulation platform mainly includes interaction modules, dynamics modules, core modules, underwater scene modules, etc. The interaction module enables communication with the external environment, while the dynamics module ensures the accurate representation of underwater robot dynamics. The core module is responsible for loading the robot URDF model and the main scene model. Lastly, the underwater scene module is employed to visualize underwater scenes and obstacles.

We converted the RoboDact robot fish model into a URDF file using SolidWorks and successfully imported it into UWSim. Additionally, we designed a 3D fish-shaped dynamic obstacle using 3D MAX and imported its corresponding osg file into UWSim. The simulated underwater environment provided by UWSim was utilized for our experiments. Both the BUV model and the fish-shaped dynamic obstacle were loaded into the simulation environment, allowing us to conduct our tests effectively.

**Fig. 9.**
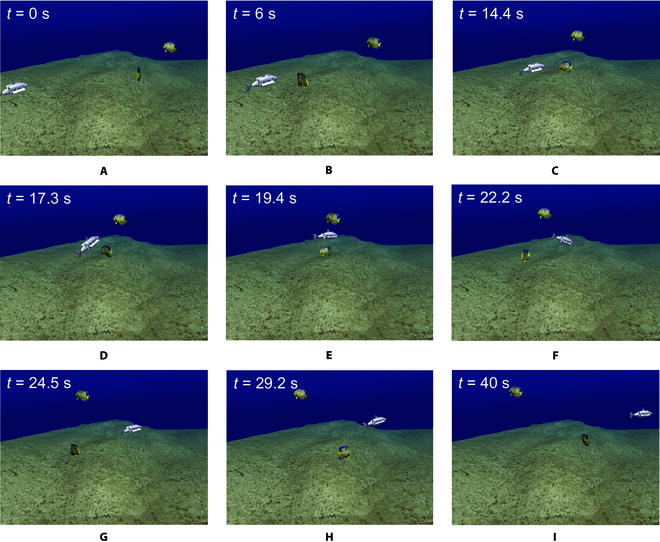
Sequence of semiphysical simulation screenshots in UWSim.

**Fig. 10.**
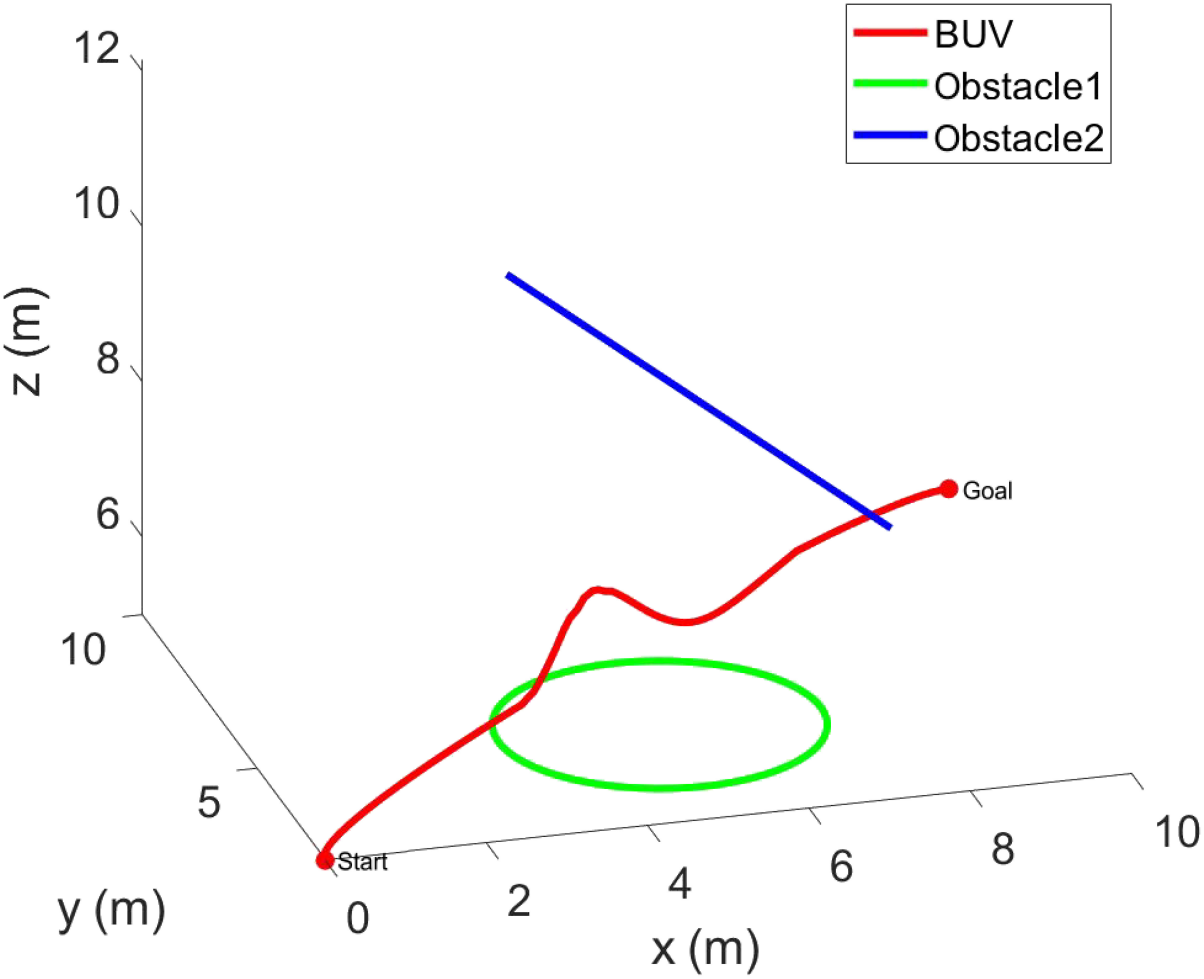
Trajectories of BUV and obstacle fishes in UWSim.

The start position and end position of the BUV are (0,2,5) and (10,10,5.5) in meters. We set up 2 fish-shaped dynamic obstacles between the start and the end points, which move in a circular trajectory and a linear trajectory, respectively. After planning a global trajectory for the BUV through the Bezier curve global trajectory planning method, the BUV is controlled by a fuzzy PID controller to track the trajectory for motion in the simulation environment. When an obstacle fish is encountered, real-time local trajectory planning is performed by the IPPO algorithm to avoid the obstacle and return to the global trajectory. The simulation results are shown in Fig. 9. The trajectories of BUV and obstacle fishes are shown in Fig. 10.

**Fig. 11.**
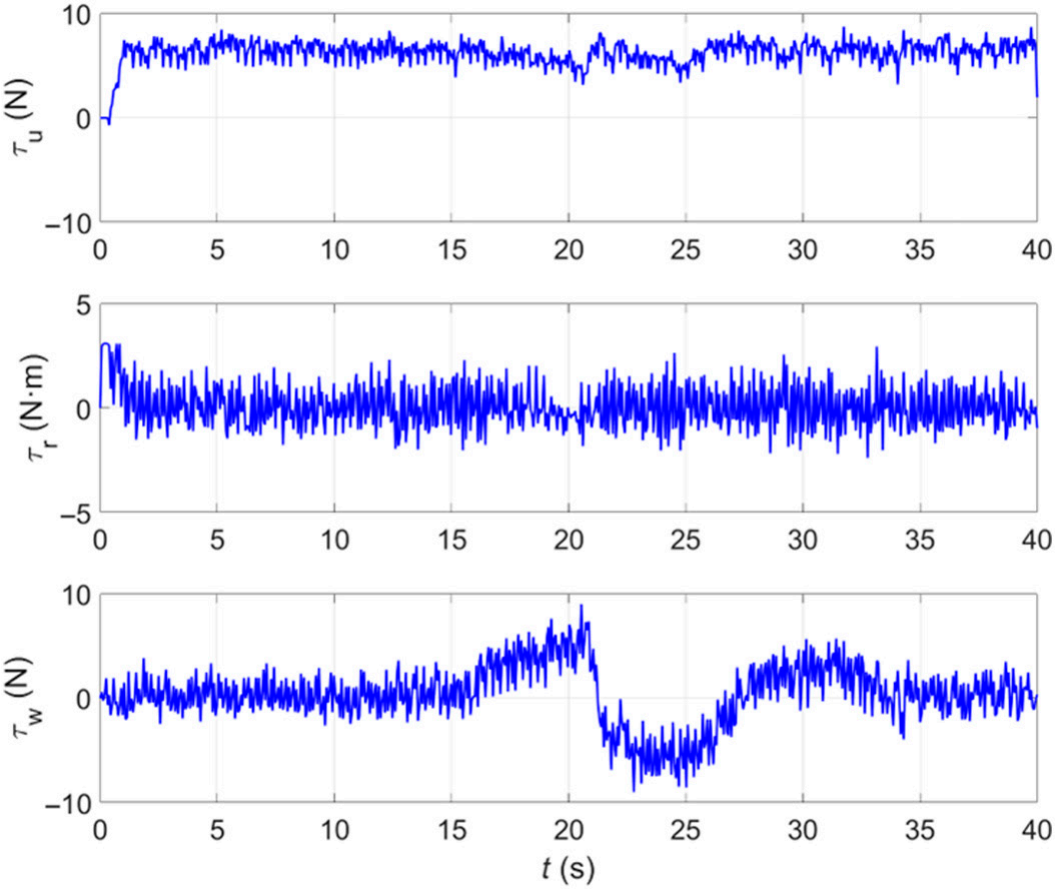
Time evolution of control signals.

**Fig. 12.**
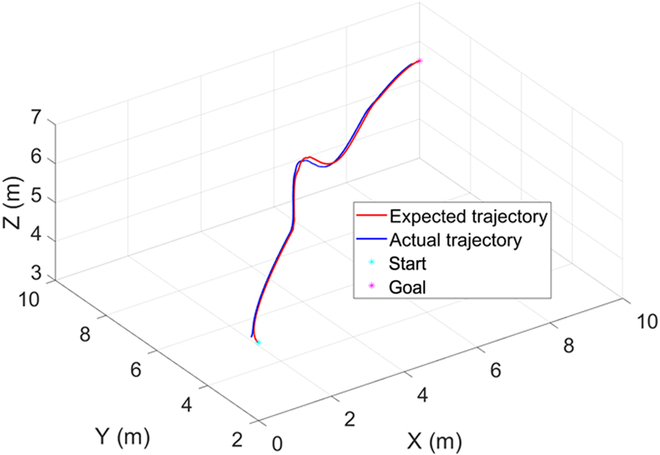
Comparison between expected trajectory and actual trajectory.

**Fig. 13.**
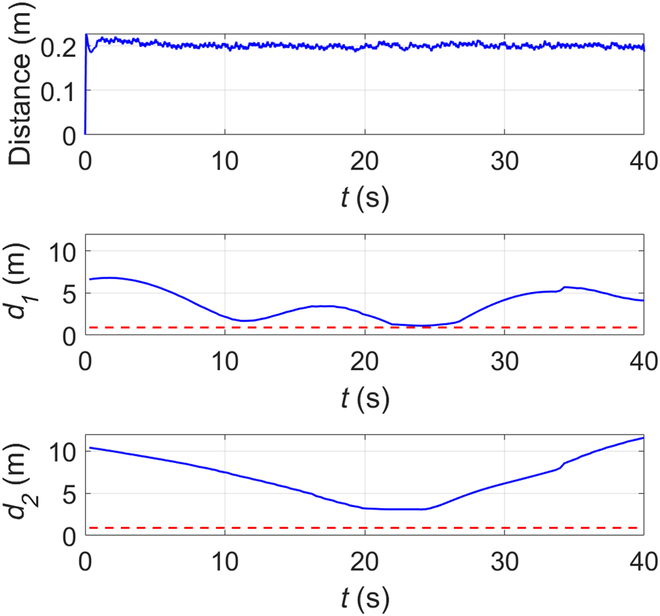
Time evolution of the tracking error and the distance between BUV and 2 obstacles.

As can be seen from the figure, at 0 s, the BUV is located at the starting position and starts to advance along the Bezier curve global trajectory. At 14.4 s, a fish-shaped obstacle is detected to prepare for real-time local trajectory planning. From 17.3 to 24.5 s, the bionic underwater robot advances on the local trajectory to bypass the fish-shaped obstacle. At 29.2 s, it returns to the global trajectory and reaches the end position at 40 s. It can be seen that the method of this paper still has satisfactory performance in the uwsim simulation environment. Figures 11 to 13 give the outputs of the controller at various moments on the body-fixed thrust and torque (on surge, heave, and yaw), the actual trajectory of the BUV compared with the desired trajectory, the amount of tracking error variation, and the distance between BUV and obstacles. Notice that the control inputs are propulsive force and torque in the simulation. While the actual control inputs of the RoboDact is the paremeters of the waves in its pair of pectoral and one caudal fins, and the relationship between them is given by Eq. 6. In Fig. 13, we set the BUV to maintain a distance of 0.2 m from the current trajectory target point, which is much smaller than the size of the RoboDact and therefore acceptable. In the case of external interference, the BUV maintains the distance to the target point well with a small error. Therefore, it can be seen that the controller has good accuracy and anti-interference ability. The red dashed line in Fig. 13 shows the safety distance of the BUV, which is 0.9 m. In Fig. 13, *d*_1_, *d*_2_ are the distances of the BUV from barrier fish 1 and barrier fish 2, respectively. The BUV keep a safe distance from the 2 barrier fishes during trajectory tracking. Thus, it can be concluded that the fuzzy PID controller in this paper can effectively control the BUV to track the desired trajectory with good accuracy and stability.

## Discussion

In this research paper, we propose a trajectory planning method that combines Bezier curves and the IPPO algorithm. The Bezier curve is initially employed to plan a global trajectory, while the IPPO algorithm is responsible for local trajectory planning when the BUV encounters obstacles during its movement. This enables the BUV to bypass obstacles and quickly return to the global trajectory. Through experiments conducted in a multidynamic obstacle environment and comparative analyses with the A* and RRT algorithms, we demonstrate the effectiveness of our proposed method and highlight its advantages in terms of trajectory length, planning time, and trajectory smoothness. Furthermore, we validate our approach through semiphysical simulation experiments on the RoboDact platform within the UWSim simulation environment, achieving satisfactory results.

In the future, we intend to conduct bionic underwater robot physics experiments to implement the trajectory planning and tracking control methods proposed in this paper on the RoboDact prototype. Furthermore, we plan to delve deeper into the research on the trajectory tracking control algorithm for bionic underwater robots, with the aim of enhancing the accuracy of trajectory tracking control. These efforts will enable us to further refine and improve the application of our methods in real-world scenarios.

## Data Availability

The data used to support the findings of this study are available from the corresponding author upon request.

## References

[1] Cong Y, Fan B, Hou D, Fan H, Liu K, Luo J. Novel event analysis for human-machine collaborative underwater exploration. Pattern Recogn. 2019;96(S2): Article 106967.

[2] An R, Guo S, Yu Y, Li C, Awa T. Multiple bio-inspired father–son underwater robot for underwater target object acquisition and identification. Micromachines. 2021;13(1):25. 35056190 10.3390/mi13010025 PMC8781731

[3] Li J, Ma J, Liu Z, Li X, Ji Y, Li S. Design of an underwater autonomous Inspection robot. In: *2022 Global Conference on Robotics, Artificial Intelligence and Information Technology (GCRAIT*); 2022. p. 27–31.

[4] Xu P, Zheng J, Liu J, Liu X, Wang X, Wang S, Guan T, Fu X, Xu M, Xie G, et al. Deep-learning-assisted underwater 3D tactile tensegrity. Research. 2023;6:0062. 36930813 10.34133/research.0062 PMC10013964

[5] Sfakiotakis M, Lane DM, Davies JBC. Review of fish swimming modes for aquatic locomotion. IEEE J Ocean Eng. 1999;24(1):237–252.

[6] Cao Q, Wang R, Zhang T, Wang Y, Wang S. Hydrodynamic modeling and parameter identification of a bionic underwater vehicle: RobDact. Cyborg Bionic Syst. 2022;2022: Article 9806328. 36285303 10.34133/2022/9806328 PMC9494701

[7] Simons DG, Bergers MMC, Henrion S, Hulzenga JIJ, Jutte RW, Pas WMG. A highly versatile autonomous underwater vehicle with biomechanical propulsion. In*: OCEANS 2009-EUROPE*; 2009. p. 1–6.

[8] Zhang S, Qian Y, Liao P, Qin F, Yang J. Design and control of an agile robotic fish with integrative biomimetic mechanisms. IEEE/ASME Trans Mech. 2016;21(4):1846–1857.

[9] Wang S, Wang Y, Wei Q, Tan M, Yu J. A bio-inspired robot with undulatory fins and its control methods. IEEE/ASME Trans Mech. 2017;22(1):206–216.

[10] Wang R, Wang S, Wang Y, Cai M, Tan M. Vision-based autonomous hovering for the biomimetic underwater robot–RobCutt-II. IEEE Trans Ind Electron. 2019;66(11):8578–8588.

[11] Cai M, Wang S, Wang Y, Wang R, Tan M. Coordinated control of underwater biomimetic vehicle–manipulator system for free floating autonomous manipulation. IEEE Trans Syst Man Cybern Syst. 2021;51(8):4793–4803.

[12] Sani M, Wang J. Research onpath and trajectory planning of underwater robot based on PID algorithm. Intell Comput Appl. 2020;10(8):206–211.

[13] Filaretov VF, Yukhimets DA, Mursalimov ES, Scherbatyuk AF, Tuphanov IE. Some marine trial results of a new method for AUV trajectory motion control. In: *2014 Oceans - St. John’s*; 2014. p. 1–6.

[14] Wang Y, Wang S, Tan M, Zhou C, Wei Q. Real-time dynamic Dubins-helix method for 3-D trajectory smoothing. IEEE Trans Control Syst Technol. 2015;23(2):730–736.

[15] Murthy K, Rock S. Spline-based trajectory planning techniques for benthic AUV operations. In: *2010 IEEE/OES Autonomous Underwater Vehicles*; 2010. p. 1–9.

[16] Zeng Z, Sammut K, He F, Lammas A. Efficient path evaluation for AUVs using adaptive B-spline approximation. In: *2012 Oceans*; 2012. p. 1–8.

[17] Li Y, Jiang Y, Zhang G, Chen P. AUV recovery path planning method considering geometrical constraints. Robot. 2015;37:478–485.

[18] Wang R, Jiang T, Bai G, Wang Y, Wang S, Tan M. Stepwise cooperative trajectory planning for multiple BUVs based on temporal–spatial Bezier curves. IEEE Trans Instrum Meas. 2023;72:1–14.37323850

[19] Gan W, Cai C, Li C, Wang H. A Trajectory planning method of autonomous underwater vehicles based on repulsive field model prediction. In: *2022 34th Chinese Control and Decision Conference (CCDC)*; 2022. p. 4671–4676.

[20] Wang C, Wei L, Wang Z, Song M, Mahmoudian N. Reinforcement learning-based multi-AUV adaptive trajectory planning for under-ice field estimation. Sensors. 2018;18(11):3859–3859. 30424017 10.3390/s18113859 PMC6263807

[21] Yang J, Xi M, Wen J, Li Y, Song HH. A digital twins enabled underwater intelligent internet vehicle path planning system via reinforcement learning and edge computing. Digit Commun Netw. 2022;In Press, Corrected Proof;S2352864822000967.

[22] Hadi B, Khosravi A, Sarhadi P. Deep reinforcement learning for adaptive path planning and control of an autonomous underwater vehicle. Appl Ocean Res. 2022;129(17): Article 103326.

[23] Wang R, Wang S, Wang Y, Wei Q. Way-point tracking control for a biomimetic underwater vehicle based on backstepping. In: *2016 35th Chinese Control Conference (CCC)*; 2016. p. 5970–5975.

[24] Oh SR, Sun J. Path following of underactuated marine surface vessels using line-of-sight based model predictive control. Ocean Eng. 2010;37(2):289–295.

[25] Wang R, Wang S, Wang Y, Tang C. Path following for a biomimetic underwater vehicle based on ADRC. In: *2017 IEEE International Conference on Robotics and Automation (ICRA)*; 2017. p. 3519–3524.

[26] Chen J, Zhu H, Zhang L, Sun Y. Research on fuzzy control of path tracking for underwater vehicle based on genetic algorithm optimization. Ocean Eng. 2018;156:217–223.

[27] Zhang C, Cheng P, Du B, Dong B, Zhang W. AUV path tracking with real-time obstacle avoidance via reinforcement learning under adaptive constraints. Ocean Eng. 2022;256(7): Article 111453.

[28] Wang N, Gao Y, Zhang X. Data-driven performance-prescribed reinforcement learning control of an unmanned surface vehicle. IEEE Trans NeurNetw Learn Syst. 2021;32(12):5456–5467. 10.1109/TNNLS.2021.3056444 33606641

[29] Wang N, Zhang Y, Ahn CK, Xu Q. Autonomous pilot of unmanned surface vehicles: Bridging path planning and tracking. IEEE Trans Veh Technol. 2022;71(3):2358–2374.

[30] Cui R, Yang C, Li Y, Sharma S. Adaptive neural network control of AUVs with control input nonlinearities using reinforcement learning. IEEE Trans Syst Man Cybern Syst. 2017;47(6):1019–1029.

[31] Wang N, Gao Y, Zhao H, Ahn CK. Reinforcement learning-based optimal tracking control of an unknown unmanned surface vehicle. IEEE Trans NeurNetw Learn Syst. 2021;32(7):3034–3045. 10.1109/TNNLS.2020.3009214 32745008

[32] Zhang T, Wang R, Wang Y, Cheng L, Wang S, Tan M. Design and locomotion control of a Dactylopteridae-inspired biomimetic underwater vehicle with hybrid propulsion. IEEE Trans Autom Sci Eng. 2022;19(3):2054–2066.

[33] Fossen TI. Guidance and control of ocean vehicles [dissertation]. [Trondheim (Norway)]: University of Trondheim; 1994.

[34] Wang R, Wang S, Wang Y, Tan M, Yu J. A paradigm for path following control of a ribbon-fin propelled biomimetic underwater vehicle. IEEE Trans Syst Man Cybern Syst. 2019;49(3):482–493.

[35] Ma R, Wang Y, Wang R, Wang S. Development of a propeller with undulating fins and its characteristics. In: *2019 IEEE International Conference on Real-time Computing and Robotics (RCAR)*; 2019. p. 737–742.

[36] Zhi Q, Mizumoto M. PID type fuzzy controller and parameters adaptive method. Fuzzy Sets Syst. 1996;78(1):23–35.

[37] Prats M, Pérez J, Fernández JJ, Sanz PJ. An open source tool for simulation and supervision of underwater intervention missions. In: *2012 IEEE/RSJ International Conference on Intelligent Robots and Systems*; 2012. p. 2577–2582.

